# Cellular heterogeneity and stem cells of vascular endothelial cells in blood vessel formation and homeostasis: Insights from single-cell RNA sequencing

**DOI:** 10.3389/fcell.2023.1146399

**Published:** 2023-03-21

**Authors:** Taku Wakabayashi, Hisamichi Naito

**Affiliations:** ^1^ Department of Ophthalmology, Osaka University Graduate School of Medicine, Osaka, Japan; ^2^ Wills Eye Hospital, Thomas Jefferson University, Philadelphia, PA, United States; ^3^ Department of Vascular Physiology, Kanazawa University Graduate School of Medical Science, Kanazawa, Ishikawa, Japan

**Keywords:** vascular endothelial cells (VECs), heterogeneity, angiogenesis, single-cell RNA sequencing, vascular endothelial stem cells, clonal expansion, CD157/Bst1, pathological angiogenesis

## Abstract

Vascular endothelial cells (ECs) that constitute the inner surface of blood vessels are essential for new vessel formation and organ homeostasis. ECs display remarkable phenotypic heterogeneity across different organs and the vascular tree during angiogenesis and homeostasis. Recent advances in single cell RNA sequencing (scRNA-seq) technologies have allowed a new understanding of EC heterogeneity in both mice and humans. In particular, scRNA-seq has identified new molecular signatures for arterial, venous and capillary ECs in different organs, as well as previously unrecognized specialized EC subtypes, such as the aerocytes localized in the alveolar capillaries of the lung. scRNA-seq has also revealed the gene expression profiles of specialized tissue-resident EC subtypes that are capable of clonal expansion and contribute to adult angiogenesis, a process of new vessel formation from the pre-existing vasculature. These specialized tissue-resident ECs have been identified in various different mouse tissues, including aortic endothelium, liver, heart, lung, skin, skeletal muscle, retina, choroid, and brain. Transcription factors and signaling pathways have also been identified in the specialized tissue-resident ECs that control angiogenesis. Furthermore, scRNA-seq has also documented responses of ECs in diseases such as cancer, age-related macular degeneration, Alzheimer’s disease, atherosclerosis, and myocardial infarction. These new findings revealed by scRNA-seq have the potential to provide new therapeutic targets for different diseases associated with blood vessels. In this article, we summarize recent advances in the understanding of the vascular endothelial cell heterogeneity and endothelial stem cells associated with angiogenesis and homeostasis in mice and humans, and we discuss future prospects for the application of scRNA-seq technology.

## 1 Introduction

Vascular endothelial cells (ECs) constitute the inner cellular lining of all blood vessels, including arteries, veins and capillaries, and are therefore essential for maintaining life. Dysfunction of ECs is associated with diverse pathologies, such as cancer, ischemic diseases, neurodegeneration, infection, and inflammation (Cai and Harrison, 2000; Hida et al., 2016; Teuwen et al., 2020; Marziano et al., 2021). Heterogeneity of vascular ECs refers to their differences and variabilities in terms of cellular morphology, gene expression, function, metabolism, and proliferative potential under physiological and pathological conditions (Minami and Aird, 2005; Aird, 2007a; Aird, 2007b; Eelen et al., 2015; Naito et al., 2020a; Goveia et al., 2020; Kalucka et al., 2020). ECs exhibit heterogeneity both within a given tissue (intra-tissue/organ heterogeneity) and across different tissues (inter-tissue/organ heterogeneity) (Potente and Mäkinen, 2017).

**FIGURE 1:**
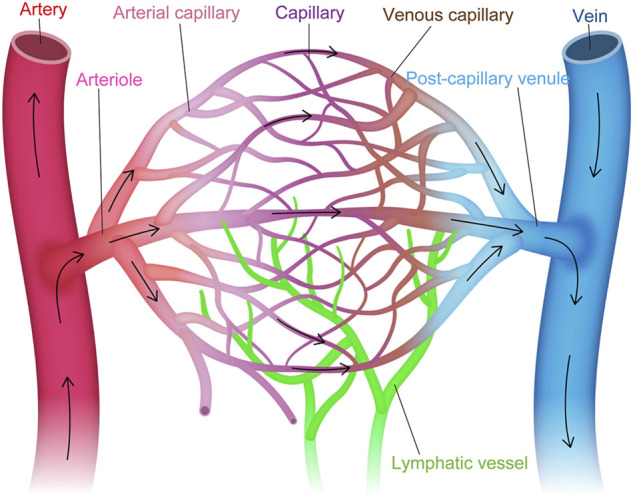
Vascular endothelial cell heterogeneity within the vascular tree. Structural and molecular heterogeneity of vascular endothelial cells (ECs) within the vascular tree, including artery, arteriole, arterial capillary, capillary, venous capillary, post-capillary venule, and vein (i.e., intra-tissue heterogeneity).

Intra-tissue heterogeneity refers to the heterogeneity of ECs within the vascular tree, including arteries, veins and capillaries (Aird, 2012) (Figure 1). The structural differences that distinguish these three types of vessels have been well established by light and electron microscopy (Paz and D’Amore, 2009), while their molecular signatures have been identified by molecular biological approaches (Wang et al., 1998; You et al., 2005). For example, arteries express specific genes, such as *Ephrin B2*, *NRP1*, *Dll4*, *Alk1*, *Depp*, *Hey1*, and *Hey2*, while veins express *Eph B4*, *COUP−TFII*, and *NRP2* (Nakagawa et al., 1999; Krebs et al., 2000; Shutter et al., 2000; Seki et al., 2003; Shin and Anderson, 2005). However, conventional approaches for investigating intra-tissue heterogeneity in ECs were limited to a focus on the differences in anatomical location, morphology, molecular signature, and structure of the blood vessels in each organ.

By contrast, the inter-tissue heterogeneity of ECs is associated with the specific functions of organs and tissues. For example, liver sinusoidal ECs participate in liver metabolism, detoxification of pathological agents, and synthesis of coagulation factor VIII (Aird, 2007b). Glomerular ECs in the kidney are crucial for glomerular filtration, while in the lung, capillary ECs in the alveolus contribute to gas exchange and regulation of blood pressure by producing angiotensin-converting enzyme (Aird, 2007a). In the brain and retina, ECs play an essential role in the formation of the blood-brain and retinal barriers (Zlokovic, 2008; Klaassen et al., 2013; Sweeney et al., 2019). These organ-specific characteristics of ECs are induced and maintained by the surrounding microenvironment and are responsible for the observed inter-tissue heterogeneity (Gifre-Renom et al., 2022). However, most studies that have investigated inter-tissue heterogeneity were based on a bulk approach involving isolation of the whole complement of ECs in an organ using pan-endothelial markers, such as CD31 (PECAM1) and VE-cadherin (Nolan et al., 2013; Coppiello et al., 2015; Khan et al., 2019). Therefore, ECs that constitute a small proportion of the vascular ECs may have been overlooked or were undetectable.

Heterogeneity also exists in terms of the proliferative potential of ECs during angiogenesis, a process of neovessel formation. Early histopathologic studies have suggested that new vessel sprouts originate from veins and post-capillary venules (Folkman, 1982). Much attention was paid to VEGF/VEGF receptors, Ephrin/Eph, angiopoietin-Tie, and Notch signaling in early studies focusing on the mechanisms of angiogenesis (Potente et al., 2011; Ricard et al., 2021). However, recent studies using single cell culture techniques, genetic lineage tracing, and side population methods have focused on the origin of the specific ECs responsible for angiogenesis, and have revealed the presence of specialized ECs that have the potential for clonal expansion and can contribute to new vessel sprouting (Naito et al., 2012; 2016; Basile and Yoder, 2014; Wakabayashi et al., 2018).

More importantly, different subtypes of ECs can now be profiled by single-cell RNA sequencing (scRNA-seq), an emerging and rapidly expanding technology that can profile individual cell transcriptomes (Chavkin and Hirschi, 2020; Kalucka et al., 2020). The scRNA-seq technique has overcome many limitations of the conventional approaches, such as sequential analysis of gene expression (SAGE), microarrays, or RNA sequencing using bulk samples, where transcriptomes from different cell types are aggregated and analyzed together. In this article, we summarize recent advances in the understanding of EC heterogeneity in mice and humans through scRNA-seq technology, and we discuss the prospects for its expanded application.

## 2 Single cell RNA sequencing of endothelial cells

Recent advances in scRNA-seq include the identification of new and rare EC subtypes, such as aerocytes (Gillich et al., 2020), the hierarchy of ECs (Rohlenova et al., 2020), novel marker genes for EC subtypes (Kalucka et al., 2020), and vascular biology mechanisms underlying organ homeostasis and diseases (Goveia et al., 2020; Becker et al., 2022). These new findings are expected to further improve our understanding of the mechanisms of vasculature formation and homeostasis, while also contributing to the development of new therapies for blood vessel–related diseases.

### 2.1 The first step in scRNA-seq (endothelial cell isolation for scRNA-seq)

The first and probably the most important step in scRNA-seq is the acquisition of viable single-cell suspensions from the tissue of interest. Various methods have been reported, including organ perfusion, laser capture microdissection, selective attachment to culture dishes, manual sorting, magnetic-activated cell sorting (MACS), and fluorescence-activated cell sorting (FACS) (Ball et al., 2002; Beijnum et al., 2008; Meyer et al., 2016; Cabral et al., 2018). Among these methods, we propose enzymatic digestion and mechanical disaggregation, followed by FACS, for obtaining reproducible and high-quality data (Naito et al., 2020b).

For enzymatic digestion, we adopt a three-step digestion with dispase, crude collagenase, and type II collagenase. The time of enzymatic digestion and the degree of mechanical force (shaking) depends on the size, softness, and age of the target organ (Naito et al., 2020b; Wakabayashi et al., 2022). Under-digestion of the tissue may result in a smaller acquisition of single cell suspensions, while over-digestion can induce cell death and less viable cells. After enzymatic dissociation, we immunostain cells for CD31 and CD45. The ECs defined as CD31^+^CD45^−^ cells can be sorted by FACS. Recent studies have explored digestion methods for each tissue, including liver, heart, lung, kidney, brain, and retina (Wakabayashi et al., 2018; 2022; Lee et al., 2019; Manolopoulou et al., 2019; Naito et al., 2020b).

### 2.2 Single cell RNA sequencing

**FIGURE 2:**
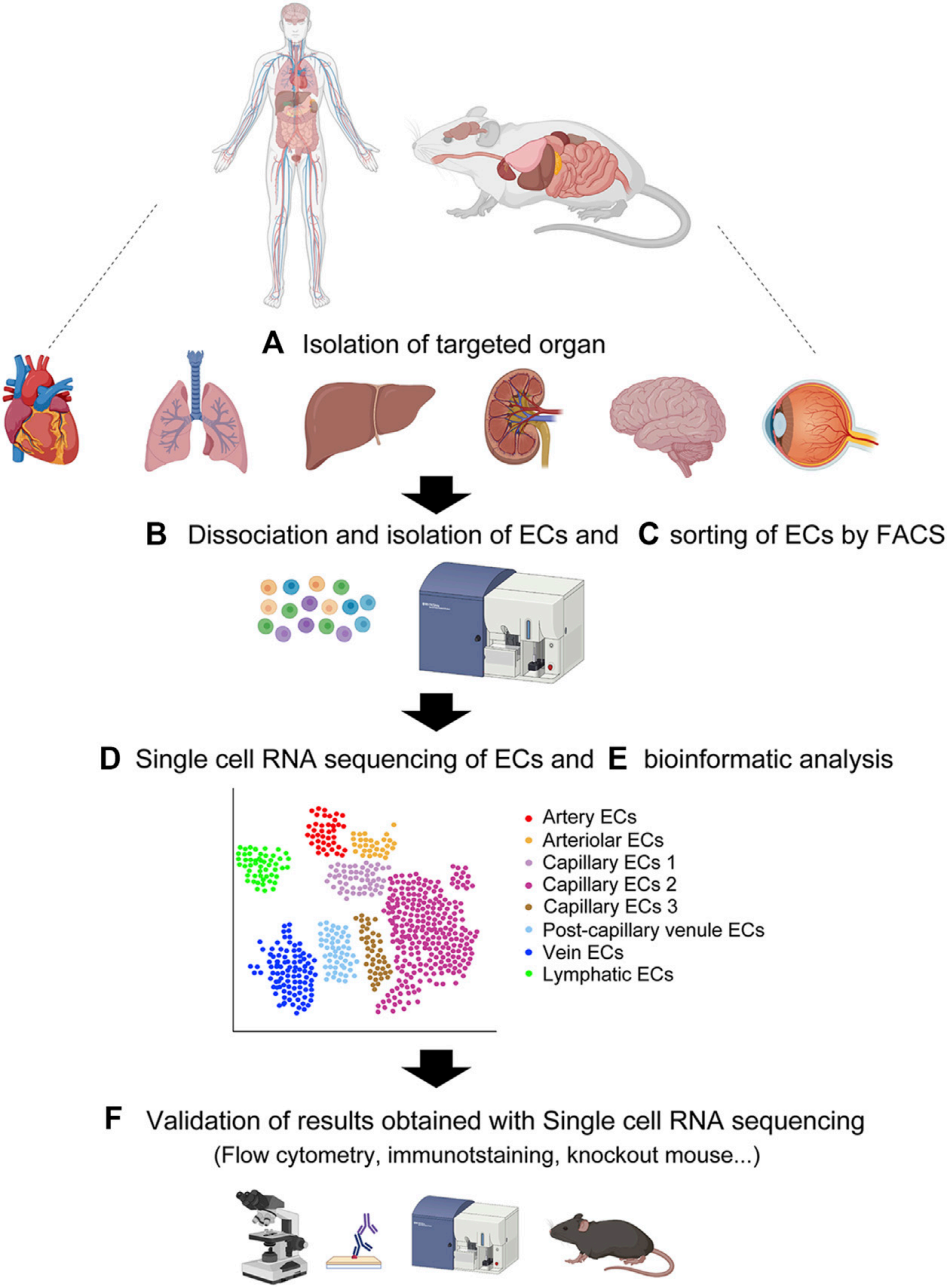
Overview of single-cell RNA sequencing. **(A)** Isolation of target organ. **(B)** Creating single cell suspension by enzymatic digestion. **(C)** Flow cytometric sorting of CD31-positive CD45-negative vascular endothelial cells. **(D)** Single cell RNA sequencing of the single cell suspension. **(E)** Bioinformatic analysis of the single cell transcriptomes. **(F)** Validation of results obtained with single cell RNA sequencing by various approaches. Created with BioRender.com.

Following sorting of the single-cell suspensions of ECs, each cell is captured for scRNA-seq (Figure 2). The droplet-based approach using 10X Genomics Chromium (10X) and the plate-based approach with the Smart-seq2 method are two common scRNA-seq platforms. The details of each method are available elsewhere (Picelli et al., 2013; Wang et al., 2021). In both methods, each EC is lysed under conditions that preserve RNA, and the recovered RNA is then reverse-transcribed into complementary DNA (cDNA). Next, the cDNA is amplified by PCR to generate sufficient material for sequencing. The amplified and tagged cDNA from every EC is then pooled and sequenced by next-generation sequencing. Finally, single-EC datasets that consist of heterogeneous EC subtypes are assembled, assessed for quality and variability, and specifically analyzed by bioinformatics programs that allow pre-processing (quality control, normalization, data correction, dimensionality reduction, and feature extraction) and downstream analyses of scRNA-seq data using R and/or Python (McCarthy et al., 2017; Hwang et al., 2018; Chen et al., 2019). The scRNA-seq bioinformatic analysis provides clustering of heterogeneous EC subtypes with different gene expression profiles through various cell clustering methods with software tools that partition cells into subtypes (Satija et al., 2015; Kiselev et al., 2017).

## 3 Organ-specific vascular endothelial cell heterogeneity in healthy organs

The organ-specific heterogeneity of ECs is induced early during embryonic development and persists throughout adulthood to maintain diverse organ functions. Features of the surrounding tissue-specific microenvironment, such as neighboring cells, hemodynamics, organ-dependent growth factors, and metabolism, play an important role in maintaining this EC heterogeneity. Interestingly, each vascular EC subtype is characterized by distinct gene signatures associated with its respective role Table 1.

**TABLE 1:** Marker genes in organ-specific vascular subtypes.

Tissue	Subtypes	Mouse	Human	References
Multiple organs	Pan-ECs	*Pecam1, Kdr, Cdh5, Cldn5, Emcn*	*PECAM1, KDR, CDH5, FLT1, TEK, CLDN5, VWF*	Nakagawa et al. (1999); Krebs et al. (2000); Shutter et al. (2000); Seki et al. (2003); Shin and Anderson (2005)
	Artery	*Ephrin B2, NRP1, Dll4, Alk1, Depp, Hey1, Hey2*	*GJA4, GJA5, HEY1, GATA2, CXCR4, SOX17, MECOM*	
	Vein	*Eph B4, COUP−TFII, NRP2*	*ACKR1, PLVAP, NR2F2*	
	VESC	*Cd157, Abcg2, Abcb1a*	*ABCG2, ABCB1*	Naito et al. (2012); Naito et al. (2020b); Wakabayashi et al. (2013); Wakabayashi et al. (2018); Wakabayashi et al. (2022)
Lung	Alveolar capillary (aerocyte)	*Apln, Car4, Ednrb*	*FOXP2, TBX2, EDNRB, SPON2, CLEC4E, S100A4, EMC, APLN, PRKG*	Ellis et al. (2020); Gillich et al. (2020); Kalucka et al. (2020); Schupp et al. (2021); Wakabayashi et al. (2018); Naito et al. (2020a)
	Alveolar capillary (gCap)	*Aplnr*	*BTNL9, FCN3, CD14, CD36, GPI-HBP1, IL7R, VWF, PLVAP, PTPRB*	
	Artery	*Mpg*, *Gja5, Fbln2, Hey1*	*BMX*, *SEMA3G*, *LTBP4*, *FBLN5*, *SERPINE2*, *NOS1*, *PDE3A*, *GJA5*, *GJA4*, *EFNB2*, *SOX17*, *DKK2*, *DLL4*, *HEY1*	
	Vein	*CD200*, *Vwf, Slc6a2*	*NR2F2*, *VCAM1*, *ACKR1, ACTN1*	
Kidney	Glomerular EC	*Pi16, Plat, Ehd3, Cyb4b1, Tspan7, Lpl, Gata5, Tbx3*	*SEMA3G, CLDN5*	Dumas et al. (2020); Dumas et al. (2021); Young et al. (2018)
	Cortical EC	*Igfbp3, Npr3*		
	Medullary EC	*Igfbp7, Cd36, Igf1, Cryab, Aqp1, Ifi27l2a*		
Brain	Artery	*Mgp, Cytl1, Bmx, Hey1, Gkn3*	*BMX, EFNB2, VEGFC, SCN2A*	Vanlandewijck et al. (2018); Kalucka et al. (2020); Garcia et al. (2022); Winkler et al. (2022); Yang et al. (2022)
	Vein	*Lcn2, Tmbs10, Icam1, Ackr1*	*ACKR1, TSHZ2, ADGRG6*	
	Capillary	*Cxcl12, Spock2, Rgcc, Slc16a1*	*MFSD2A, TFRC SLC16A1, SLC7A5, SLC38A5, RGCC, SRARP*	
	Tip cells	*Piezo2, Scn1b, Kcne3, Kcna5, Pcdh17, Madcam1, Mcam, Clec1a, Sirpa, Cmklr1, Serpine1/Pai, Plaur, Adm, Angpt2, Smoc2, Pde4b, Ppm1j/Pp2c-zeta, Noxo1, Hecw2*	*PLAUR, LAMB1*	
Retina	Artery	*Bmx, Hey 1*	*BMX, EFNB2*	Sabbagh et al. (2018); Lee et al. (2021)
	Vein	*Nr2f2, Nrp2, Gm5127*	—	
	Tip cells	*Plaur, Angpt2, Lcp2, Cxcr4, Apln, Kcne3*, *Mcam*, *Lamb1*, *Trp53i11, Esm1*	*PLAUR, LAMB1*	
Choroid (eye)	Artery	*Eln*, *Mgp, Gkn3*, *Cxcl12*, *Aqp1*, *Emcn*	*GJA4, GJA5, HEY1, FBLN2, SEMA3G*	Rohlenova et al. (2020); Voigt et al. (2019)
	Vein	*Vwf, Eln*, *Mgp*	*DARC*, *MMRN1*	
	Capillary	*Plvap, Flt1*	*CA4*, *PLVAP*, *SIPR3 SPARC*, *PCDH12*, *RGCC*	
	Post-capillary venule	*Selp*, *Ackr1*	*DARC*, *MMRN1*	
Liver	Artery	*Clu, Plac8, Lrg1*	—	Kalucka et al. (2020); Inverso et al. (2021); Koch et al. (2021); Halpern et al. (2018); MacParland et al. (2018); Gómez-Salinero et al. (2022)
	Portal vein	*Cd34, Ephb2, Ly6a*	*RAMP3, IFITM1, INMT, DNASE1L3, LIFR*	
	LSEC (Zone 1, periportal)	*Efnb2, Ltbp4, Dll4, Msr1*	*MGP, SPARCL1 TM4SF1, CLEC14A, IDI, IGFBP7*	
	LSEC (Zone 2, midlobular)	*Stabilin-1, Stabilin-2 CD206, Lyve-1, Ecm1*	*LYVE1, STAB2, CCL14, CLEC1B, FCN2, S100A13*	
	LSEC (zone 3, pericentral)	*Wnt2, Wnt9b, Rspo3*		
	Central vein	*Lhx6, Wnt2, Fgfr2, Cdk1*	*Wnt9b, Rspo3*	
Heart	Artery	*Fbln5*, *Hey1*, *Mecom, Apln, Dll4, Notch1, Cxcr4, Cx40, Igfbp3*	*HEY1*, *DKK2*, *EFNB2, SEMA3G, DLL4*	Kalucka et al. (2020); Koenig et al. (2022)
	Vein	*Mgp*, *Cfn*, *Bgn*, *Vwf*	*ACKR1, NR2F2*	
	Capillary	*Kdr*, *Endou*	*BTNL9*, *CD36, RGCC, CA4*	

### 3.1 Lung

#### 3.1.1 Lung vascular anatomy

The lung has two different circulatory pathways. The pulmonary circulation participates in gas exchange, whereas the bronchial circulation delivers oxygen and nutrients to the bronchial walls. In the pulmonary circulation, the pulmonary arteries carry deoxygenated blood to the alveoli. The alveolar capillaries play an important role in blood gas exchange by adding oxygen from the alveoli to the blood and transferring carbon dioxide from the blood to the air in the alveoli. The pulmonary veins then return the newly oxygenated blood to the left atrium of the heart.

#### 3.1.2 Inter-tissue endothelial heterogeneity in the lung

Every alveolar capillary EC contains angiotensin-converting enzyme, whereas this enzyme is present in only 10% of systemic capillary ECs. The alveolar ECs are a major site of conversion of angiotensin I to the active vasoconstrictor form angiotensin II and therefore play a role in regulating vascular tone (Balyasnikova et al., 2005). The pulmonary ECs also secrete nitric oxide, prostacyclin, endothelin-1, serotonin, and thromboxane (Aird, 2007b). Recent scRNA-seq studies on lung-specific ECs have identified unique characteristics, such as an enrichment of Forkhead box F1 (Foxf1) network (Kalucka et al., 2020) and upregulation of the genes involved in cAMP metabolism, suggesting that pulmonary ECs depend on cAMP to maintain the integrity of the endothelial barrier (Sayner, 2011). Pulmonary ECs also show high expression of MHC class II genes, indicating a role in immune responses against airborne pathogens (Kalucka et al., 2020).

**FIGURE 3:**
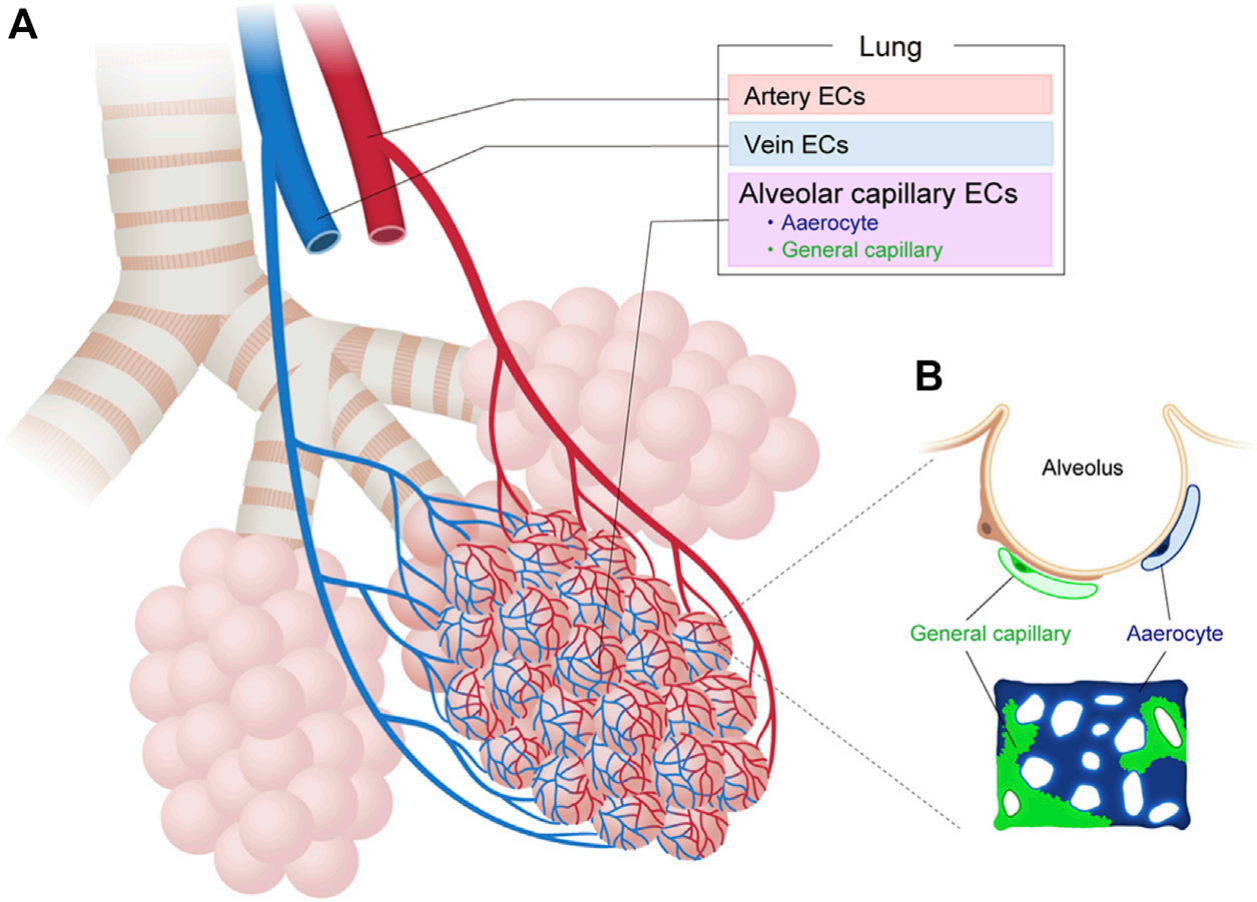
Vascular endothelial cell heterogeneity in the lung. **(A)** Vascular endothelial cells (ECs) in the lung can be classified into artery, vein, and capillary ECs based on their structure and molecular differences. **(B)** Alveolar capillary ECs are further classified into aerocytes and general capillary ECs.

Recent scRNA-seq analysis of adult mouse pulmonary ECs have also identified two molecularly distinct subtypes of alveolar ECs in an apparently homogeneous capillary vessel (Figure 3). The first EC subtype, which the researchers termed the “aerocyte” or “Car4 EC”, was marked by *Apln* and/or *Car4* and represented a unique subtype specialized for gas exchange and leukocyte trafficking in the alveoli (Ellis et al., 2020; Gillich et al., 2020). The other cell type, termed gCap for “general capillary,” was marked by *Aplnr* and specialized in the regulation of vasomotor tone. The gCap also served as a stem/progenitor cell in capillary repair and homeostasis. Both cell types likely interact with each other. Aerocytes strongly express ligands (*Kitl* and *Apln*) that can bind to the corresponding receptors (*Kit* and *Aplnr*) on gCaps. Conversely, gCap cells strongly express ligands (*Edn1* and *Vegfa*) that can bind to the corresponding receptors (*Ednrb* and *Kdr*) on aerocytes. Both EC types were found to be conserved between mouse and human lungs but neither was identified in turtle or alligator lungs, indicating that these ECs may have arisen during the evolution of the mammalian lung (Gillich et al., 2020; Schupp et al., 2021).

The scRNA-seq analysis of human lung ECs from 73 individuals confirmed the presence of aerocytes in humans, as identified by their expression of the transcription factors *FOXP2* and *TBX2*, the endothelin receptor (*EDNRB*), the pattern recognition receptors *SPON2* and *CLEC4E*, and signaling mediators, such as *CHRM2, EDA*, *PRKG*, and S100A4 (Schupp et al., 2021). Aerocytes are also characterized by their potential to degrade prostaglandins (*HPGD*), their lack of endothelial-specific Weibel-Palade bodies, and the absence of expression of genes such as *PTPRB*, *CD93*, *THBD*, *ANO2*, *LIFR*, *CRIM1*, *CYYR1*, *PALMD, GNA14*, *MECOM* and *LEPR* that are otherwise expressed in all other ECs. Conversely, human gCaps can be distinguished by their expression of genes related to innate immune responses (e.g., *BTNL9*, *FCN3*, *CD14*, and *BTNL8*), transcytosis of low-density lipoprotein cholesterol and other lipids (e.g., *CD36* and *GPI-HBP1*), and expression of cytokine receptors (e.g., *IL7R* and *IL18R1*). The ratio of aerocytes to gCap ECs is typically 0.48:1 in humans. Previously indistinguishable subtypes have also been identified among venous ECs, including pulmonary–venous ECs (*COL15A1*-negative) that are localized to the lung parenchyma and systemic–venous ECs (*COL15A1-*positive) that are localized to the bronchial vasculature and the visceral pleura (Schupp et al., 2021). These new findings have improved our understanding of the structure, function, and maintenance of gas exchange and the function of alveoli in mouse and human lungs.

#### 3.1.3 Intra-tissue heterogeneity in the lung

Recent scRNA-seq analysis has also revealed EC heterogeneity within the mouse lung, characterized by at least four distinct ECs with different gene expression profiles, namely, artery ECs (that express *Mpg*, *Gja5, Fbln2,* and *Hey1*), capillary ECs (that express *Car4*, *Fibin*, *Cyp4b1*, *Ednrb*), other capillary ECs (that express *Gpihbp1*, *Sema3c*, *Cadm1*, and *Hilpda*), and vein ECs (that express *CD200*, *Vwf,* and *Slc6a2*) (Kalucka et al., 2020).

In humans, arterial ECs were identified by their expression of *BMX*, *SEMA3G*, *LTBP4*, *FBLN5*, *SERPINE2*, *NOS1*, *PDE3A*, *GJA5*, *GJA4*, *EFNB2*, *SOX17*, *DKK2*, *DLL4*, and *HEY1* (Schupp et al., 2021). These genes are associated with tight and gap junctions, the extracellular matrix that contributes to wall elasticity and strength, protease inhibitors, signaling molecules, the nitric oxide pathway involved in vascular tonus, and transcription factors that maintain the function and identity of arteries. Capillary ECs were identified by expression of *CA4* (orthologous to mouse *Car4*) and *CYB5A* related to gas exchange, *PRX* and *SPARC* that maintain structure, and *AFF3* and *MEIS1* that are related to transcription regulation. Capillary ECs were further divided into aerocytes and gCaps.

Venous ECs were identified through their expression of the canonical transcription factor *NR2F2* (*COUP-TFII*), *VCAM1*, *ACKR1* and *ACTN1*, which are associated with transcription factors, diapedesis of leukocytes, transcytosis, and membrane proteins, respectively. Venous ECs contained two distinct populations, pulmonary-venous and systemic-venous ECs.

### 3.2 Kidney

#### 3.2.1 Kidney vascular anatomy

The kidney is critical for regulating the composition and volume of body fluids. The renal artery enters the kidney and divides into segmental arteries, followed by branching into interlobar arteries (Molema and Aird, 2012). The interlobar arteries give rise to arcuate arteries, cortical radiate arteries, and afferent arterioles. The afferent arterioles branch into glomerular capillaries and the glomerular capillaries, in turn, branch into a second arteriole type, the efferent arteriole. The efferent arterioles branch into the peritubular capillaries and descending vasa recta. Peritubular capillaries reabsorb most of the water and solutes entering the kidney. The descending vasa recta feed the next capillary networks, and these capillaries drain into ascending vasa recta, eventually returning the water and solutes to the renal vein.

#### 3.2.2 Inter-tissue endothelial heterogeneity in the kidney

Recent scRNA-seq by Barry et al. and Dumas et al. has revealed that each vessel in the kidney contains ECs with different gene expression profiles and functions. Several studies have reported the presence of unique transcription factors in the kidney, including *Tbx3*, *Pbx1*, *Gata5*, and *Prdm1*, that are essential for the structure and function of glomerular ECs in mice (Barry et al., 2019; Dumas et al., 2020). Genetic deletion of Tbx3 in ECs resulted in glomerular hypoplasia, microaneurysms, and regressed fenestrations that led to glomerular fibrosis.

Kidney ECs express gene sets involved in activation of interferon signaling, raising the possibility that this may play a role in EC homeostasis of the healthy kidney (Jourde-Chiche et al., 2019; Dumas et al., 2020; Kalucka et al., 2020). Peritubular capillary ECs in the cortex strongly express *Igfbp3* and *Npr3*, whereas medullary renal ECs strongly express *Igfbp7* and *Cd36* (Dumas et al., 2021).

#### 3.2.3 Intra-tissue endothelial heterogeneity in the kidney

**FIGURE 4:**
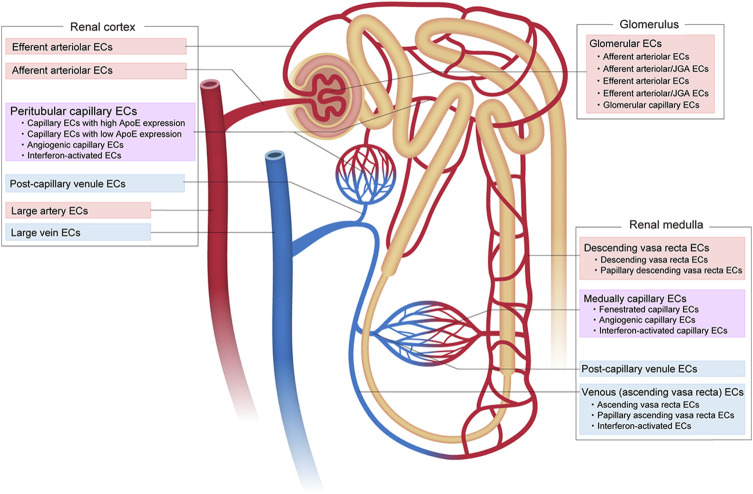
Vascular endothelial cell heterogeneity in the kidney. Vascular endothelial cells (ECs) in the kidney show diverse heterogeneity based on their structure, location, complex function, and molecular signature.

Recent scRNA-seq of the adult mouse kidney by Dumas et al. has revealed at least 24 distinct ECs with different gene expression profiles (Figure 4). These include five subtypes of glomerular ECs, nine subtypes of cortical renal ECs, and 10 subtypes of medullary ECs (Dumas et al., 2020; Dumas et al., 2021). The kidney glomerulus is responsible for the filtration of blood that forms the primary urine. The subtypes of glomerular ECs include: 1) afferent arteriolar ECs, 2) ECs from the terminal portion of the afferent arterioles associated with the juxtaglomerular apparatus (JGA), 3) fenestrated capillary glomerular ECs, 4) ECs expressing a mixture of genes associated with efferent and arteriole JGA, and 5) efferent arteriolar ECs. The afferent arteriolar ECs express *Edn1*, *Alox12*, *Gja4*, *Kcnn4*, and *S1pr1*, the afferent arterioles associated with JGA express *Gja5* and *Cd300lg*, the capillary ECs express *Smad6*, *Lpl*, *Ehd3*, *Abcc4*, *Nostrin*, *Sema5a*, and *Scn7a*, the efferent arterioles associated with JGA express *Slc26a10*, *Ptprr*, and *Gas5*, and the efferent arteriolar ECs express *Calca*, *Klf4*, *Slc6a6*, and *Cryab*. Each gene in each EC subtype has a role in kidney function and is an identity (marker). For example, Gja5, also known as connexin 40, controls renin release by regulating the communication between ECs and the granular cells in the JGA (Kurtz et al., 2007).

In the renal cortex, peritubular capillaries provide oxygen and nutrients, while contributing to the reabsorption and secretion of solutes and water (Molema and Aird, 2012). Nine subtypes of cortical renal ECs are recognized: 1) large arterial ECs that express *Eln, Ltbp4*, *Mgp*, *Gja5*, *Slc8a1*, *Cldn5*, and *Kcnn4;* 2) afferent arteriolar ECs that express *Edn1*, *Gja4*, *Alox12*, *Bmp4*, *Cldn5*, *Slc18a1*, *S1pr1*, *Kcnn4,* and *Cxcl12*; 3) efferent arterioles that express *Calca*, *Klf4*, *Slc6a6* and *Cryab*; 4) capillary ECs that strongly express *ApoE*; 5) capillary ECs that show low *ApoE* expression; 6) post-capillary venule ECs that express *Kdr*, *Nr2f2*, *Tnxb* and *Jup*; 7) vein ECs that express *Nr2f2*, *CD9*, *Gas6* and *Plvap*; 8) capillary ECs with an angiogenic phenotype that express *Gpihbp1*, *Col4a1*, *Esm1*, *Col4a2*, *Trp53i11*, *Aplnr*, *Apln*, *Plk2* and *Fscn1*; and 9) capillary ECs that exhibit an IFN-response phenotype and express *Isg15* and *Ifit* family genes.

The renal medulla plays a role in urine concentration. Ten subtypes of medullary ECs have been identified, as follows: 1) arteriole ECs that express *Sox17*, *Gja4* and *Fbln5*; 2) descending vasa recta ECs that express *Slc14a1*, *Scin*, *Aqp1*, *Cldn5*, *Slc5a3* and *Crip1*; 3) ECs from the papillary portion of the descending vasa recta that express *S100a4*, *S100a6*, *Fxyd5*, *Akr1b3*, and *Nrgn*; 4) fenestrated capillary ECs that express *Plpp3* and *Cd36*; 5) post-capillary venule ECs that express *Kdr*, *Nr2f2*, *Tnxb* and *Jup*; 6) vein-like ECs in the ascending vasa recta that express *Gas6*, *Tek*, and *Fxyd6*; 7) vein-like ECs in the papillary portion of the ascending vasa recta that express *S100a6*, *Fxyd5*, *Akr1b3*, *Aldoa*, *Nrgn*, *Crip1*, *Gapdh* and *Ldha*; 8) capillary ECs with an angiogenic phenotype that express *Gpihbp1*, *Col4a1*, *Esm1*, *Col4a2*, *Trp53i11*, *Aplnr*, *Apln, Plk2* and *Fscn1*; 9) capillary ECs with an IFN-response phenotype that express *Isg15* and *Ifit* family genes, and 10) venous ECs with an IFN-response phenotype that also express *Isg15*, and *Ifit* family genes.

In humans, scRNA-seq revealed glomerular ECs that highly express *SEMA3G* and *CLDN5*, ECs in the descending vasa recta that express *KDR and PTPRB*, and ECs in the ascending vasa recta that express *PLVAP* (Young et al., 2018).

Overall, renal ECs display significant structural and functional heterogeneity. In addition, in response to acute dehydration, the kidney increases water reabsorption, concentrates the urine, and reduces glomerular filtration. Kidney EC scRNA-seq has revealed that the medullary ECs physiologically adapt to hypertonicity by upregulating transporters required for the accumulation of cytoprotective organic osmolytes and genes for oxidative phosphorylation.

### 3.3 Brain

#### 3.3.1 Brain vascular anatomy

The internal carotid arteries and vertebral arteries are responsible for the anterior and posterior circulation of the brain, respectively. The internal carotid arteries branch into the anterior and middle cerebral arteries that supply the medial and lateral parts of the frontal and parietal lobes, basal ganglia, internal capsule corpus, and callosum. The posterior circulation is supplied by vertebral arteries that fuse at the level of the pons to form the basilar artery, which then divides into the posterior cerebral arteries. The basilar artery joins the blood circulation from the internal carotids in the arterial ring, known as the circle of Willis. The anterior inferior cerebellar artery arises from the basilar artery, while the posterior inferior cerebellar artery arises from the vertebral arteries to supply the cerebellum. These arteries are connected to the brain capillaries and then to the venous system (e.g., the cerebral veins) and the dural venous sinuses that drain into the jugular vein.

#### 3.3.2 Inter-tissue endothelial heterogeneity in the brain

ECs that form the brain vasculature comprise unique morphological and functional units, known as neurovascular units, that maintain the blood-brain barrier (BBB) protecting the brain from neurotoxicity. The characteristics of brain ECs include a lack of fenestration and the expression of unique tight junction complexes, ATP-binding cassette (ABC) transporters, and transporters involved in the transport of docosahexaenoic acid (*Mfsd2a*), amino acids (*Slc7A5 and Slc3A2*), and glucose (*Glut1/Slc2A1*) (Garlanda and Dejana, 1997; Profaci et al., 2020). Recent scRNA-sequencing of mouse brain ECs has revealed further brain EC–specific characteristics, such as enrichment of genes typically responsible for neuronal function, including neurotransmitter transport, synapse organization, and axon development (Sabbagh et al., 2018; Jambusaria et al., 2020; Kalucka et al., 2020; Paik et al., 2020). Brain EC–specific transcription factors that maintain the integrity of the BBB, such as Forkhead box Q1 (*Foxq1*) and Forkhead box F2 (*Foxf2*), have also been identified by scRNA-seq. Several genes, such as *Pglyrp1*, *Lcn2*, and *Tmem100*, are expressed specifically in brain ECs but not in other organs (Kalucka et al., 2020).

#### 3.3.3 Intra-tissue endothelial heterogeneity in the brain

scRNA-seq of the developing mouse brain has identified a new tip-cell marker gene responsible for protein secretion, as well as receptors and membrane and intracellular proteins, such as *Piezo2*, *Scn1b*, *Kcne3*, *Kcna5*, *Pcdh17*, *Madcam1*, *Mcam*, *Clec1a*, *Sirpa*, *Cmklr1*, *Serpine1/Pai*, *Plaur*, *Adm, Angpt2*, *Smoc2*, *Pde4b*, *Ppm1j/Pp2c-zeta*, *NADPH oxidase organizer-1 (Noxo1)*, and *Hecw2* (Sabbagh et al., 2018). scRNA-seq studies of the adult mouse brain have demonstrated a transcriptional zonation in the brain vasculature, characterized by gradual phenotypic changes along the anatomical arteriovenous axis (Vanlandewijck et al., 2018). Nine distinct brain EC subtypes have been identified in the mouse, namely, those of the large artery, shear-stress artery, arterial capillary, capillary, venous capillary, large vein, and choroid plexus, as well as interferon-activated ECs (Kalucka et al., 2020). The large artery ECs express *Fbln5* and *Cytl1*, arterial capillary ECs express *Tgfb2* and *Glul*, capillary ECs express *Rgcc*, venous capillary ECs express *Car4* and *Tfrc*, large vein ECs express *Lcn2,* and choroid plexus capillary ECs express *Plvap* and *Esm1*. The expression of *Vwf* and *Vcam1* was highest in ECs from large arteries and large veins (Gustavsson et al., 2010).

scRNA-seq of human brain has also revealed transcriptional zonation in the brain arteriovenous axis, but the genes are very different between species, with only a small subset conserved (∼10%) between mice and humans (Garcia et al., 2022; Winkler et al., 2022; Yang et al., 2022). For example, *VEGFC*, *BMX*, and *EFNB2* are expressed in both human and mouse arterioles and *MFSD2A* and *TFRC* in both human and mouse capillaries, whereas *TSHZ2* and *LRRC1* are both expressed in human venules but not in mouse ECs. *ANO2* is expressed in capillaries/venules in human but not in mouse ECs. Garcia et al. also found specific expression of *TSHZ2* in human venule zonation, and EC–specific expression of the metallothioneins *MT1E* and *MT2A* in the human cortex vasculature but not in the mouse.

### 3.4 Retina

#### 3.4.1 Retinal vascular anatomy

The retina has one of the highest metabolic demands of tissues that are responsible for vision. The retina is supplied by the central retinal artery derived from the ophthalmic artery and its branches (Kur et al., 2012). The human central retinal artery branches into retinal arteries and arterioles. The arterioles then bifurcate to form smaller arterioles and branch into three capillary beds: the superficial, intermediate, and deep capillary plexuses around the macula (Campbell et al., 2017; Yu et al., 2019). The superficial capillaries are predominantly located in the ganglion cell layer (GCL), the intermediate capillaries are located above the inner nuclear layer (INL), and the deep capillaries are located below the INL. The intermediate capillaries decrease in density toward the periphery, as the retina thins, and are no longer detectable in the periphery, resulting in two capillary plexuses, the superficial and the deep one (Lavia et al., 2020).

Another radially-oriented capillary network, consisting of the radial peripapillary capillaries, is present near the optic disc (Henkind, 1967; Spaide et al., 2015). A capillary-free zone exists around the retinal arteries and arterioles because of the high oxygen concentration in these vessels. The venous system (post-capillary venules and veins) collects the blood from the capillaries and drains venous blood into the superior ophthalmic vein and the cavernous sinus.

#### 3.4.2 Inter-tissue endothelial heterogeneity in the retina

Similar to brain ECs, retinal ECs constitute a neurovascular unit that maintains the inner blood-retinal barrier (BRB) to protect the retina from neurotoxicity (Klaassen et al., 2013). Therefore, retinal ECs lack fenestration and express tight junction complex proteins, including ZO-1, Occludin, and Claudins, as well as several other proteins, including ABC transporters, Mfsd2a, amino acid transporters (e.g., Slc7A5 and SlC3A2), and Glut1/SlC2A1 (Yemanyi et al., 2021).

#### 3.4.3 Intra-tissue endothelial heterogeneity in the retina

**FIGURE 5:**
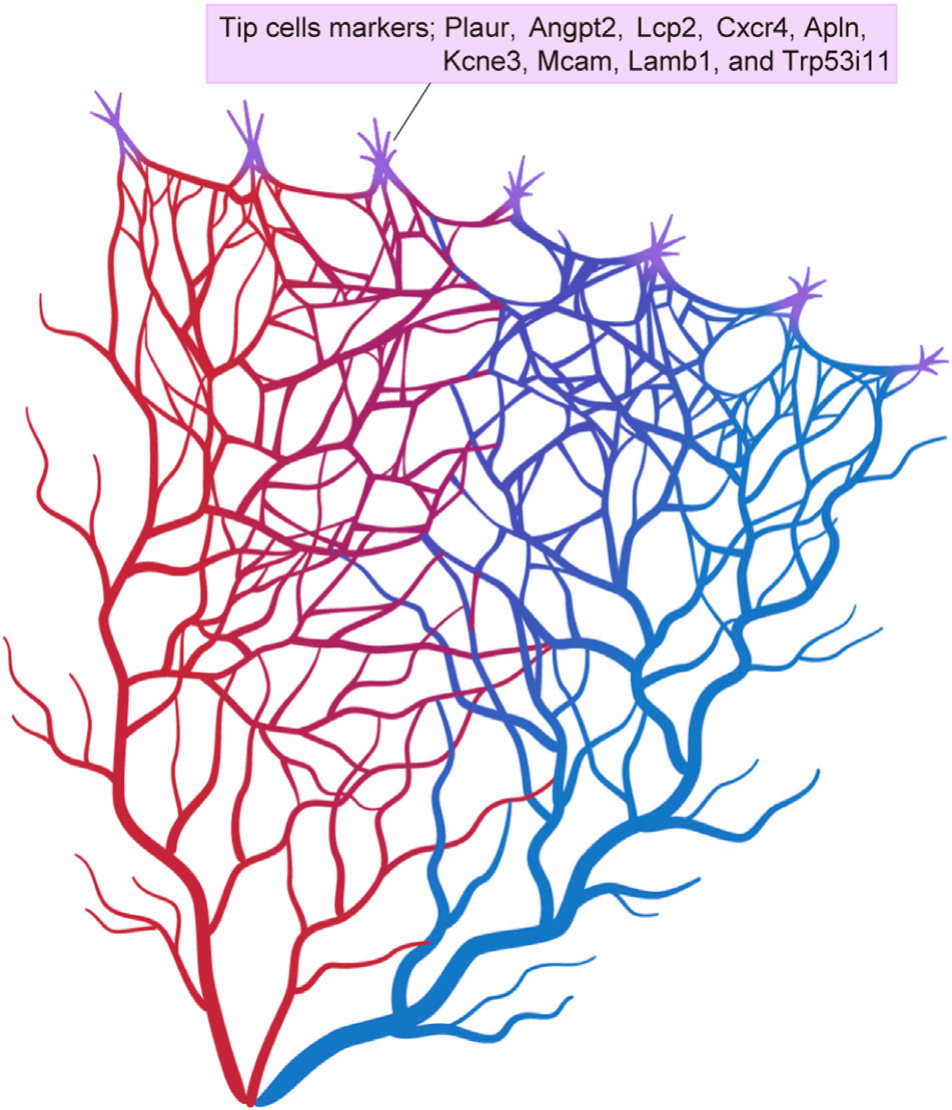
Tip cell markers. Single-cell RNA sequencing has identified various different tip cell markers in the brain and retina.

scRNA-seq of retinal ECs has focused in particular on the heterogeneity of tip cells during development. In addition to the previously known tip cell markers, which include *Plaur, Angpt2, Lcp2, Cxcr4, Apln,* and *Kcne3*, recent scRNA-seq has identified new marker genes, including *Mcam*, *Lamb1*, and *Trp53i11* (Sabbagh et al., 2018) (Figure 5). These markers have been validated by *in situ* hybridization (ISH) in whole mount mouse retinas. Gene expression profiles of the tip cells have revealed that these are more closely related to arterial ECs than to venous ECs. Another study has shown that the tip cells that guide the deep retinal vasculature (D-tip cells) are distinct from the tip cells that guide the superficial retinal vascular plexus (Zarkada et al., 2021). D-tip cells have a unique transcriptional signature that includes high TGF-β signaling, and they begin to acquire blood-retina barrier properties. A study by Zarkada et al. also indicated a contribution of stage-specific tip cells in deep retinal vascular development and suggested that TGF-β signaling may improve retinal vascularization in ischemic retinal diseases.

### 3.5 Choroid (eye)

#### 3.5.1 Choroidal vascular anatomy

The choroid is located between the retina and sclera in the eye and is composed of a vascular network and pigmented stroma. The choroid supplies oxygen and nutrients to the photoreceptors and is supplied by the long and short posterior ciliary arteries and the anterior ciliary arteries. The choroidal vasculature is divided into three layers, namely, Haller’s layer, Sattler’s layer, and the choriocapillaris (Sattler, 1876; Hayreh, 1975; Nickla and Wallman, 2010). Haller’s layer (the outer choroid) includes non-fenestrated large arteries and veins, while Sattler’s layer (the inner choroid) is composed of medium and small arterioles that supply the choriocapillaris. The choriocapillaris (the innermost choroid) is a highly anastomosed and fenestrated capillary network. Venous drainage of blood from the choriocapillaris occurs mainly *via* four vortex veins that ultimately merge with the superior and inferior ophthalmic veins.

#### 3.5.2 Inter-tissue endothelial heterogeneity in the choroid

The choroid is associated with the pathogenesis of age-related macular degeneration, the most common cause of blindness in developed countries. Consequently, the choroid ECs have been well studied by scRNA-seq. A recent study of scRNA-seq of the adult mouse retinal pigment epithelium/choroid identified a high level of Indian hedgehog homolog (Ihh), which is involved in the hedgehog signaling pathway, as one of the transcriptional signatures that characterize choroidal ECs (Lehmann et al., 2020). Choroidal EC-secreted Ihh targets Gli1 positive mesenchymal cells, which mediate choroidal and retinal inflammatory responses.

#### 3.5.3 Intra-tissue endothelial heterogeneity in the choroid

In the mouse, scRNA-seq has revealed at least 3 ECs subtypes in the choroidal arteries, two in the choriocapillaris, and four in the choroidal veins (Rohlenova et al., 2020). The three subtypes of arterial ECs included large-artery ECs that express *Eln*, *Mgp,* and *Gkn3*, arteriolar ECs that express *Cxcl12*, *Aqp1*, and *Emcn*, and shear stress-induced arterial ECs that express *Pi16*. The two choriocapillaris ECs include the outer choriocapillaris ECs characterized by fenestration (*Plvap* expression) and high VEGF-pathway signaling, and the inner choriocapillaris ECs characterized by low VEGF signaling (high *Flt1*). The four venous EC subtypes included large-vein ECs (*Vwf, Eln*, *Mgp*), shear stress–induced venous ECs (*Pi16*, *Klf4*), post-capillary venule ECs (*Selp*, *Ackr1*), and venule ECs (with a mixed choriocapillaris and post-capillary venule signature).

In humans, the choroidal veins and venules strongly express the chemokine receptor *DARC*, adhesion protein *MMRN1*, and genes associated with leukocyte recruitment, including *SELE*, *DARC*, *VCAM1*, and *CCL23* (Voigt et al., 2019). Choriocapillaris ECs strongly express membrane-associated carbonic anhydrase *CA4*, fenestration-associated *PLVAP*, barrier function-associated *SIPR3* and *SPARC*, cellular adhesion *PCDH12*, and the cell cycle gene *RGCC*. Arterial ECs strongly express gap-junction *GJA4* and *GJA5*, Notch signaling *HEY1*, and the artery-enriched genes *FBLN2* and *SEMA3G*.

### 3.6 Liver

#### 3.6.1 Liver vascular anatomy

The liver receives a dual blood supply from the hepatic artery and the portal vein. The hepatic artery brings oxygenated blood to the liver, while the portal vein delivers deoxygenated, but nutrient-rich, blood to the liver from gastrointestinal organs. The two blood sources mix in the liver sinusoids, which represent a three-dimensional capillary network. The central veins and hepatic veins collect the blood and lead it ultimately to the inferior vena cava.

#### 3.6.2 Inter-tissue endothelial heterogeneity in the liver

Liver sinusoidal ECs (LSECs) display multi-functional properties, including the regulation of hepatic circulation, filtration, endocytosis, antigen presentation, leukocyte recruitment, and regeneration of hepatocytes (Shetty et al., 2018; Gracia-Sancho et al., 2021; Koch et al., 2021). The hepatic circulation is modulated by transcription factor Krüppel-like factor 2 (KLF2)-dependent release of nitric oxide (NO) by LSECs in response to shear stress (Poisson et al., 2017). Filtration is maintained by the presence of fenestrations in LSECs and the absence of a continuous basement membrane. Endocytosis is mediated by scavenger receptors, such as SR-A, SR-B (SR-B1 and SR-B2/CD-36), mannose receptor (CD206/SR-E3), SR-H (stabilin-1 and stabilin-2), and FcγRIIb (Poisson et al., 2017). Endocytosis of high-density lipoproteins (HDLs) and modified low-density lipoproteins (LDLs) by LSECs contributes to lipid homeostasis. The lysosomal activity of LSECs aids the degradation of many waste products in the blood. LSECs also present antigens to elicit T cell responses and contribute to innate and adaptive immunological responses. LSECs also function in the trafficking of leukocytes. The secretion of HGF and Wnt2 by LSECs regulates the maintenance and regeneration of hepatocytes (Ding et al., 2010). LSECs strongly express lymphatic vessel endothelial hyaluronan receptor 1 (Lyve1), VEGFR3, and stabilin-2 (Ding et al., 2010) and can be immunostained for these proteins.

scRNA-seq has revealed enrichment of the Gata family in murine liver ECs (Kalucka et al., 2020). In line with reports that Gata4 is essential for liver development (Divine et al., 2004; Watt et al., 2007), Gata4 was specifically expressed in liver ECs.

#### 3.6.3 Intra-tissue endothelial heterogeneity in the liver

**FIGURE 6:**
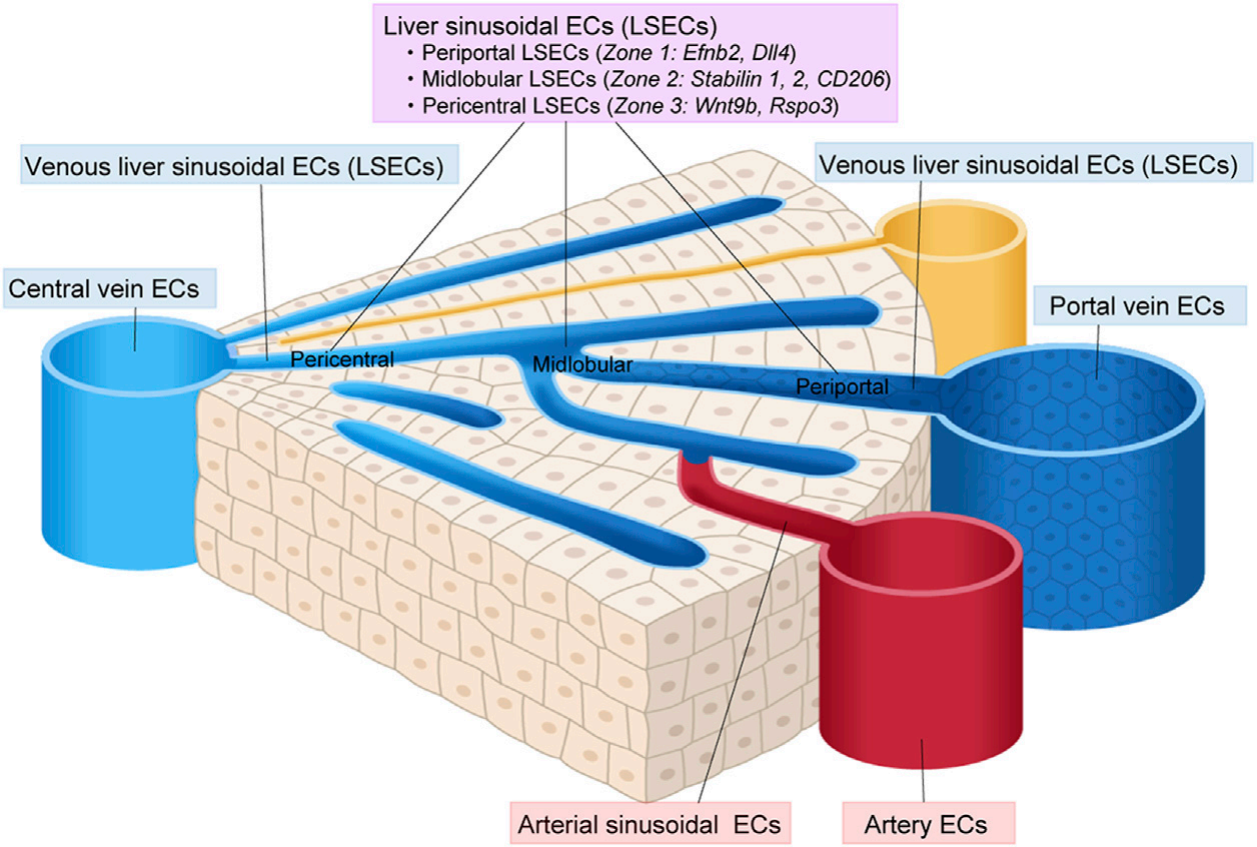
Vascular endothelial cell heterogeneity in the liver. Vascular endothelial cells (ECs) in the liver show diverse heterogeneity. Liver sinusoidal ECs (LSECs) can be classified into periportal, midlobular, and pericentral ECs.

scRNA-seq has revealed a subclustering of liver ECs into large artery ECs (that express *Clu*, *Plac8*, and *Lrg1*), arterial sinusoid ECs (that express *Efnb1*, *Gulul*, *Gja4*, and *Sox17*), sinusoid ECs (that express *Stab2* and *Kdr*), venous sinusoid ECs (that express *Thbd*), vein ECs (that express *Vwf*, *Edn1*, and *Bgn*), and proliferating ECs (that express *Stmn1* and *Cdkn1a*) (Kalucka et al., 2020). Sinusoid ECs could be further subdivided into three zone-specific subtypes based on their gene expression profiles, namely, periportal LSECs, midlobular LSECs and pericentral LESCs (Halpern et al., 2018; Inverso et al., 2021; Koch et al., 2021) (Figure 6). The periportal LSECs express *Efnb2, Ltbp4, and Dll4*. The midlobular LSECs express the most *Stabilin-1*, *Stabilin-2, Lyve-1, and CD206,* and therefore serve as scavenger cells. The pericentral LESCs secrete *Wnt2*, *Wnt9b*, and Wnt-signaling enhancer *Rspo3*, which fuel pericentral Axin2^+^ Tbx3^+^ hepatocytes (Rocha et al., 2015; Wang et al., 2015).

scRNA-seq of human liver ECs has also revealed a zonation of the LESCs similar to that seen in the mouse liver (MacParland et al., 2018; Gómez-Salinero et al., 2022). These contribute to the predominance of tyrosine kinase Tie1 phosphorylation in pericentral LESCs, resulting in a Wnt9b gradient during hepatocyte regeneration.

### 3.7 Heart

#### 3.7.1 Heart vascular anatomy

The coronary arteries branch off from the ascending aorta to supply the heart. The left main coronary artery (LMCA) and the right coronary artery (RCA) are two primary coronary arteries. The LMCA branches into the left anterior descending artery (LAD) and the left circumflex (LCx) coronary arteries to supply the anterior portion of the left ventricle, the left atrium, and the lateral/posterior-lateral aspect of the left ventricle. The LAD further branches into the obtuse marginal artery (OMA), diagonal artery, and septal perforator artery. The RCA branches into the sinoatrial nodal artery to supply the sinoatrial node, the atrioventricular nodal artery to supply the atrioventricular node, and the right posterior descending artery (PDA) and right marginal artery. The PDA supplies the posterior one-third of the interventricular septum. The coronary arteries branch into intramuscular arteries that penetrate the myocardium perpendicularly to form arterioles and capillaries that supply the cardiomyocytes. The coronary veins return deoxygenated blood from the myocardium through the coronary sinus to the right atrium.

#### 3.7.2 Inter-tissue endothelial heterogeneity in the heart

Recent scRNA-seq analysis of the mouse heart has revealed a distinct molecular signature for heart ECs, which express myosin regulatory light chain 2 (*Myl2*), myosin regulatory light chain 3 (*Myl3*), ADP-ribosylhydrolase like 1 (*Adprhl1*), alpha 2-HS glycoprotein (*Ahsg*), aquaporin 7 (*Aqp7*), xin actin-binding repeat containing 2 (*Xirp2*), myoglobin (*Mb*), sodium-coupled nucleoside transporter (*Slc28a2*), Butyrophilin-like 9 (*Btnl9*), creatine kinase, leucine rich repeats and transmembrane domains 1 (*Lrtm1*), mitochondrial 2 (*Ckmt2*), Natriuretic Peptide Receptor 3 (*NPR3*), cytokine-like 1 (*Cytl1*), *Cd36*, and fatty acid-binding protein 4 (*Fabp4*) (Feng et al., 2019; Jambusaria et al., 2020; Kalucka et al., 2020). The upregulation of *Aqp7*, *Cd36*, and *Fabp4*, which are responsible for fatty acid metabolism, is consistent with the dependance of the heart on fatty acids to generate ATP. *Npr3* and *Cytl1* are expressed by endocardial ECs, and *Cd36* and *Fabp4* are expressed by coronary vascular ECs.

#### 3.7.3 Intra-tissue endothelial heterogeneity in the heart

scRNA-seq of the mouse heart has revealed at least seven EC subtypes, as follows: artery ECs (that express *Fbln5*, *Hey1*, and *Mecom*), arterial capillary ECs (that express *Cxcl12* and *Rbp7*), capillary ECs (that express *Kdr*, *Endou*), venous capillary ECs (that express *Vcam1*, *Fmo1*, and *Fmo2*), vein ECs (that express *Mgp*, *Cfn*, *Bgn*, and *Vwf*), interferon-responsive ECs (that express *Isg15*, *Ifit3*, *Ifi203*, *Ifit1*, and *Ifit3b*), and angiogenic ECs (that express *Col4a2*, *Sparcl1*, *Aplnr*, and *Trp53i11*) (Kalucka et al., 2020).

scRNA-seq of the human heart has also revealed several EC subtypes, including arterial ECs (that express *HEY1*, *DKK2*, and *EFNB2*), venous ECs (that express *ACKR1* and *NR2F2*), capillary ECs (that express *BTNL9* and *CD36*), lymphatic ECs (that express *CCL21* and *PROX1*), and endocardial ECs (that express *NRG3* and *PCDH15*) (Koenig et al., 2022). Heterogeneity related to sex was also reported (Koenig et al., 2022).

## 4 Vascular endothelial cell heterogeneity in adult angiogenesis

Angiogenesis is the growth of new blood vessels from the existing vasculature and occurs under hypoxic and inflammatory conditions (Potente et al., 2011). Angiogenesis may occur throughout life, in both physiological and pathological circumstances in health and disease. Angiogenesis in adults is observed under many conditions, including cancer, AMD, rheumatoid arthritis, and wound healing. Therefore, cellular sources and mechanisms responsible for angiogenesis have been extensively studied. The basic process of angiogenesis includes degradation of capillary basement membrane, proliferation and migration of ECs, lumen formation, fusion and pruning of ECs, and pericyte coverage, occurring in response to angiogenic stimuli.

The reported cellular sources of new ECs during angiogenesis include bone marrow (BM)-derived circulating endothelial progenitor cells (EPCs), resident mature ECs in the pre-existing blood vessels that proliferate in a stochastic manner (random model), and specialized vascular endothelial stem cells (VESCs) that reside within the pre-existing blood vessels and proliferate in a hierarchical manner (hierarchical model) (Asahara et al., 1997; Grant et al., 2002; Wakabayashi et al., 2018; Naito et al., 2020a). Recent studies have shown that BM-derived EPCs rarely differentiate into ECs, suggesting a minor or no contribution to angiogenesis (Palma et al., 2003; Grunewald et al., 2006; Okuno et al., 2011). By contrast, endothelial colony-forming cells (ECFC) and ECs with the side population (SP) phenotype (EC-SPs), defined as CD31^+^VE-cadherin^+^CD45^−^Hoechst^low^, have been reported as vessel-resident VESCs responsible for angiogenesis (Ingram et al., 2004; Naito et al., 2012; Wakabayashi et al., 2013; Banno and Yoder, 2018). However, no unique markers of ECFCs and EC-SPs had been identified until recently, thereby limiting the recognition of VESCs. The Confetti reporter mouse line that allows the labeling of individual cells has identified clonal expansion of the EC after ischemia and EC injury (Mondor et al., 2016; Manavski et al., 2018; McDonald et al., 2018). However, whether the clonal expansion in angiogenesis occurred as hypothesized by the random model or the hierarchical model could not be distinguished by specific markers.

**FIGURE 7:**
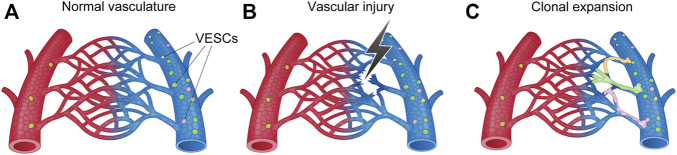
Schematic illustration of angiogenesis (i.e., new blood-vessel formation) from vascular endothelial stem cells (VESCs) by clonal expansion. **(A)** VESCs reside within the intima of the blood vessel. Mouse VESCs strongly express CD157 (Bst1), Myc, ATF3, Fosl2, Sox7, Foxp1, ABCB1a, and ABCG2. **(B)** Angiogenic stimuli activates VESCs following vascular injury, ischemia, and inflammation. **(C)** New sprouting occurs from nearby VESCs to form new blood vessels.

Our group recently identified CD157, also known as BM stromal antigen-1 (Bst1), as a marker of VESCs in mouse blood vessels that proliferate in a hierarchical manner (Wakabayashi et al., 2018) (Figure 7). These CD157-positive VESCs reside in the large blood vessels of various adult mouse organs, including liver, brain, lung, heart, limb muscle, retina, choroid, aorta, and inferior vena cava (Wakabayashi et al., 2018; 2022; Naito et al., 2020b). The proportions of VESCs among all ECs in each organ range from 1.6% to 14.9%. Genetic lineage tracing in mouse liver revealed that VESCs proliferate and act as angiogenesis-initiating cells to regenerate functional blood vessels from large vessels (portal veins) to capillaries (sinusoids) in response to acute liver injury or as part of physiological turnover (Wakabayashi et al., 2018). Single VESCs can also generate over 2000 ECs *in vitro* and can form three-dimensional functional blood vessels *in vivo* after transplantation, reflecting their stem cell properties. CD157-positive VESCs were not derived from the mesenchymal lineage or BM.

VESCs strongly express transcriptional factors, such as *Myc, ATF3, Fosl2, Sox7,* and *Foxp1*, that are known to regulate cellular proliferation and differentiation (Francois et al., 2010; Zhang et al., 2010; Wilhelm et al., 2016; McDonald et al., 2018). ABC transporters, such as ABCB1a and ABCG2, are also strongly expressed in VESCs (Wakabayashi et al., 2013; 2018). These genes may be responsible for the higher proliferative potential of CD157-positive VESCs compared with non-VESCs.

Recent scRNA-seq of choroidal ECs and lung ECs showed that genes that are enriched in VESCs, such as *CD157, ABCB1a, ABCG2,* and *ABCC4*, are highly expressed in veins and post-capillary venules (Goveia et al., 2020; Rohlenova et al., 2020). That is, among heterogenous vascular elements, including arteries, arterioles, capillaries, post-capillary venules, and veins, the VESCs are present predominantly in the veins and post-capillary venules. scRNA-seq also showed that the hierarchy of vascular ECs originates in veins and post-capillary venules that express VESC markers and that the ECs differentiate into an immature ECs, tip ECs, and then into more mature neophalanx ECs (Rohlenova et al., 2020). An early histopathological study showed that angiogenesis originates in the veins and post-capillary venules (Folkman, 1982; Ishibashi et al., 1987). Recent genetic lineage tracing also confirmed the contribution of venous ECs to angiogenesis in mice (Lee et al., 2021). Taken together, the evidence supports the possibility that VESCs present in veins and post-capillary venules with specific functional and molecular characteristics contribute to angiogenesis. In other words, the specific characteristics of VESCs may explain why angiogenesis originates in specific locations, such as veins and post-capillary venules.

Surprisingly, despite the clearly different roles of the blood vessels in each organ, the VESCs share the common marker CD157 in mice. However, we have not yet determined why CD157 should be a marker of VESCs. The CD157 molecule is a glycosyl-phosphatidylinositol (GPI)-anchored membrane protein that is thought to act independently as a receptor and enzyme (Itoh et al., 1994; Kaisho et al., 1994; Ortolan et al., 2018). As a receptor, CD157 initiates the intracellular signal transduction that results in the activation of downstream PI3K/Akt and MAPK/ERK signaling pathways that regulate cell survival, adhesion, and migration (Ortolan et al., 2018). As an enzyme, CD157 has a role in nicotinamide adenine dinucleotide (NAD^+^) metabolism and catalyzes the conversion of NAD^+^ to cyclic ADP-ribose (cADPR) and NADP^+^ to nicotinic acid adenine dinucleotide phosphate (NAADP). NAD^+^ is important for the function of various stem cells (Zhang et al., 2016). Therefore, CD157 may also, in part, influence the function of VESCs. However, we have not yet identified any apparent abnormalities in the blood vessels in CD157-knockout mice (Wakabayashi et al., 2018; 2022). Thus, genes that are involved in the key functional features of CD157-positive VESCs should be explored in future studies.

Similarly, other groups have also suggested the presence of resident VESCs and endothelial progenitors with different markers. These include EPCR (CD201 or Prcor), VE-cadherin^+^CD31^low^VEGFR2^low^IL33^+^Sox9^+^Sox18^+^, PW1/Peg3, CD117 (c-kit), and CD133 (Fang et al., 2012; Sekine et al., 2016; Yu et al., 2016; Malinverno et al., 2017; Patel et al., 2017; Lukowski et al., 2019). Potential overlap between these VESCs and CD157-positive VESCs may exist and should be investigated in the future.

## 5 Endothelial heterogeneity in disease

### 5.1 Tumor angiogenesis in cancer

Under normal physiological conditions, heterogeneous ECs in each organ have a specific role in organ homeostasis. However, in response to the development of cancer, the characteristics of these ECs change to control pathological angiogenesis, leukocyte infiltration, and vascular permeability due to hypoxia, various tumor-derived cytokines, reactive oxygen species, and epigenetic changes. The result is a transition to a more complex heterogeneity within the tumor blood vessels (Sun et al., 2017; Goveia et al., 2020; Rohlenova et al., 2020; Teuwen et al., 2021; Xie et al., 2021). In the tumor microenvironment, tumor cells and other cells secrete cytokines, such as vascular endothelial growth factor (VEGF) and basic fibroblast growth factor (FGF), thereby inducing vessel sprouting (sprouting angiogenesis) (Weis and Cheresh, 2011). During vessel sprouting, new sprouts are led at the forefront by tip ECs, followed by stalk ECs and mature ECs referred to as phalanx ECs (Welti et al., 2013).

**FIGURE 8:**
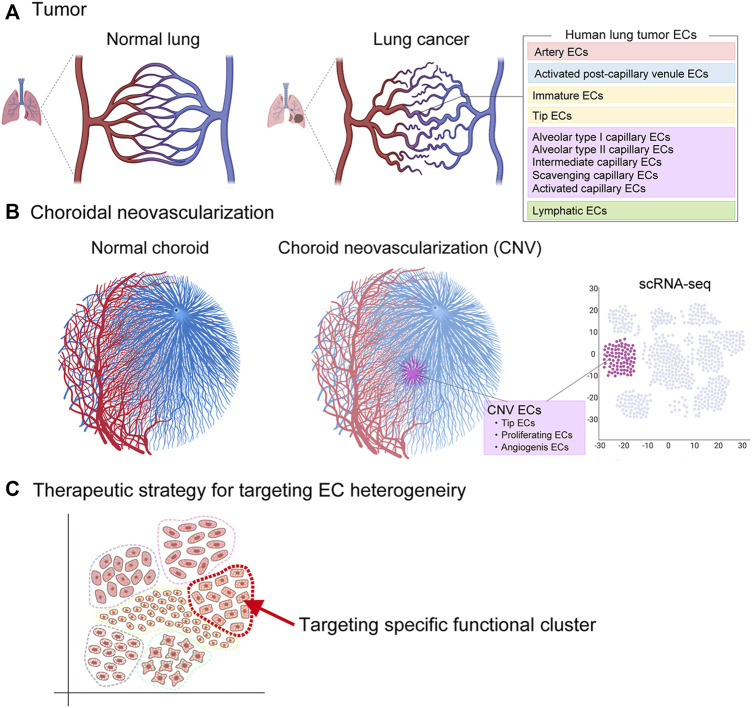
Vascular endothelial cell heterogeneity in pathological angiogenesis. Vascular endothelial heterogeneity exists within pathological vessels seen in **(A)** tumor (cancer) and **(B)** choroidal neovascularization (age-related macular degeneration). Potential therapeutic strategy to target endothelial cells responsible for pathological angiogenesis **(C)**.

Recent scRNA-seq of human lung cancers has identified phenotypically heterogeneous tumor EC phenotypes, including artery ECs, activated postcapillary venule ECs, immature ECs, tip cell ECs, alveolar type I capillary ECs, alveolar type II capillary ECs, intermediate capillary ECs, scavenging capillary ECs, activated capillary ECs, and lymphatic ECs (Goveia et al., 2020) (Figure 8). Among these ECs, the scavenging capillary ECs and activated capillary ECs represent two novel capillary EC phenotypes recently identified by scRNA-seq. Scavenging capillaries strongly express scavenging receptors and genes associated with macrophages and antigen processing, such as *CD36*, *CD68*, *MARCO*, *MSR1*, *CTSD*, and *CTSS*, while activated capillaries express EC activation markers, such as *CAV1*.

The tip EC is a conserved phenotype in both human and mouse tumors, and this may indicate the important role of sprouting angiogenesis for tumor growth in across species. Common tip EC markers include *ANGPT2*, *FSCN1*, *APLN*, *PGF*, *ADM*, *PLXND1*, *CXCR4*, and *PDGFB*. Enrichment of novel transcription factors, such as *TCF4*, *SOX4*, and *SMAD1*, has also been identified in tumor tip ECs (Goveia et al., 2020). In addition, scRNA-seq has identified a previously unrecognized EC phenotype, the so-called “breach” cell, in murine lung tumors (Goveia et al., 2020). Breach cells express tip cell markers (*Apln*, *Cxcr4*), but they also express genes related to basement-membrane breaching, such as *Itga6*, *Itgb1*, *Itgb3*, *Mmp14*, and *Hspg2*. They also play a role in the initiation of vessel sprouting by degrading the basement membrane to assist tip cells to lead the sprout.

One characteristic of tumor ECs is an increase in glycolytic metabolism, which is required to meet the great energetic requirement for sprouting angiogenesis. Although normal ECs show glycolytic activity (Quintero et al., 2006; Davidson and Duchen, 2007; De Bock et al., 2013a; De Bock et al., 2013b), tumor ECs have a far higher glycolytic rate (Annan et al., 2019). Increased glycolysis in tumor tip ECs is regulated by the glycolytic activators 6-phosphofructo-2-kinase/fructose-2,6-biphosphatase 3 (PFKFB3) and phosphofructokinase 1 (PFK1), which are important for cell migration and proliferation (De Bock et al., 2013b). Tumor ECs also exhibit increases in gene expression related to other metabolic pathways, such as the tricarboxylic acid (TCA) cycle, one-carbon metabolism, nucleotide synthesis, and oxidative phosphorylation (Rohlenova et al., 2020). Thus, metabolic targeting of ECs may have potential as a new therapeutic approach for inhibiting tumor angiogenesis and halting tumor growth. In fact, genetic ablation or pharmacologic inhibition of PFKFB3 can impair tip cell behavior and reduce sprouting, resulting in improved anti-cancer drug efficacy and decreased metastatic events in mouse tumor models (Cantelmo et al., 2016). The efficacy of metabolic targeting should be explored in the future for human cancer treatment.

scRNA-seq has also aided in the exploration of the sensitivity of specific EC phenotypes to anti-VEGF therapy in a mouse lung tumor model. Anti-VEGF treatment of ECs did not affect the components of heterogeneous EC subtypes; however, the proportion of tip cells and breach cells significantly decreased, while the proportion of postcapillary venules significantly increased. These responses indicate that tip and breach ECs in the tumor were most sensitive to VEGF blockade and the postcapillary venules least sensitive (Goveia et al., 2020). Tip cells comprise only a minority (less than 10%) of human lung tumor ECs; therefore, the majority of tumor ECs may not be affected by anti-VEGF therapy.

In addition, ECs in post-capillary venules contain a subset of VESCs. These VESCs strongly express multidrug resistance genes, so they can survive and remain in the tumor to serve as EC-supplying cells even after treatment with antiangiogenic drugs (Naito et al., 2016). The small proportion of anti-VEGF–sensitive tip cells within the tumor vasculature and the lack of susceptibility and survival of postcapillary venules may account for the resistance of tumors to antiangiogenic therapy. Future therapies should target the postcapillary venule as a promising approach to overcome treatment resistance.

### 5.2 Choroidal neovascularization in age-related macular degeneration

Age-related macular degeneration (AMD) is a major cause of blindness in patients older than 50 years (Jager et al., 2008). Two types of AMD, dry and wet, are recognized, and the wet AMD form is associated with the abnormal growth of blood vessels in the choroid of the eye (choroidal neovascularization). scRNA-seq of choroidal neovascularization in a mouse laser-induced model has identified an association of choroidal neovascularization with different EC phenotypes, including tip ECs, proliferating ECs, and ECs with signatures associated with the transition from postcapillary venule ECs to angiogenic ECs (Rohlenova et al., 2020). The tip cell markers were similar to those found in cancers.

A recent comparative study of normal choroidal ECs and ECs associated with choroidal neovascularization revealed two metabolic angiogenic targets, Aldh18a1 and Sqle, that were associated with the growth of choroidal neovascularization. Silencing of Aldh18a1 and Sqle decreased the expression of genes involved in cell proliferation and DNA replication and reduced the size of the choroidal neovascularization (Rohlenova et al., 2020). Thus, scRNA-seq has the potential to identify new therapeutic targets for treatment of AMD. Another study also found that the regulator of cell cycle gene (RGCC) was the most upregulated gene in a choroid from a human donor diagnosed with AMD. Further study is needed to investigate the significance of the increased RGCC expression (Voigt et al., 2019).

### 5.3 Endothelial cell response to acute myocardial infarction

Acute myocardial infarction (MI) is a life-threatening condition characterized by ischemic necrosis of cardiomyocytes resulting from acute coronary artery obstruction. Necrosis of cardiomyocytes triggers inflammatory cell infiltration, extracellular matrix (ECM) remodeling, angiogenesis, and fibroblast activation. The use of Confetti reporter mice showed that the cellular source of angiogenesis after MI was the proliferating ECs that originated from resident ECs *via* clonal expansion (Li et al., 2019). Some studies have reported that cardiac fibroblasts also contribute to coronary vessel formation through the mesenchymal-to-endothelial transition (MEndoT) (Ubil et al., 2014). However, a recent study using genetic lineage tracing of mesenchymal cells showed that cardiac fibroblasts do not contribute to the formation of new coronary vessels, indicating no contribution of MendoT to angiogenesis (He et al., 2017). Further studies are needed to elucidate whether the ECs are the only contributors to angiogenesis or whether other lineage cells may participate in new vessel formation.

The EC response to MI has been investigated by scRNA-seq of murine cardiac ECs 3 to 7 days after MI, which revealed an upregulation of mesenchymal markers, such as *Col1a1*, *Col3a1*, and *Serpine1*, and the downregulation of endothelial marker genes, such as *Cdh5*, suggesting that ECs undergo the endothelial to mesenchymal transition (EndMT) (Tombor et al., 2021). However, this induction of the EndMT was transient, and the EC clusters returned to the endothelial transcriptomic state at least 10 days post-MI. Although the EndMT has been implicated in cardiac fibrosis after MI (Zeisberg et al., 2007), its role in the post-infarction healing process is still not well understood and requires further study.

Another scRNA-seq analysis of murine cardiac ECs after MI showed that the EC clusters upregulated genes related to cardiac muscle morphogenesis (*Myl2*, *Myl3*, *Mb*, *Tnnt2*, *Actc1*, and *Tnni3*), indicating that cardiac ECs may switch on cardiomyogenic genes in response to MI (Li et al., 2019). The ECs clusters also strongly express *Plvap*, which plays a role in EC proliferation, and may therefore represent a new therapeutic target for promotion of cardiac repair after MI (Li et al., 2019).

## 6 Conclusion and future perspectives

**FIGURE 9:**
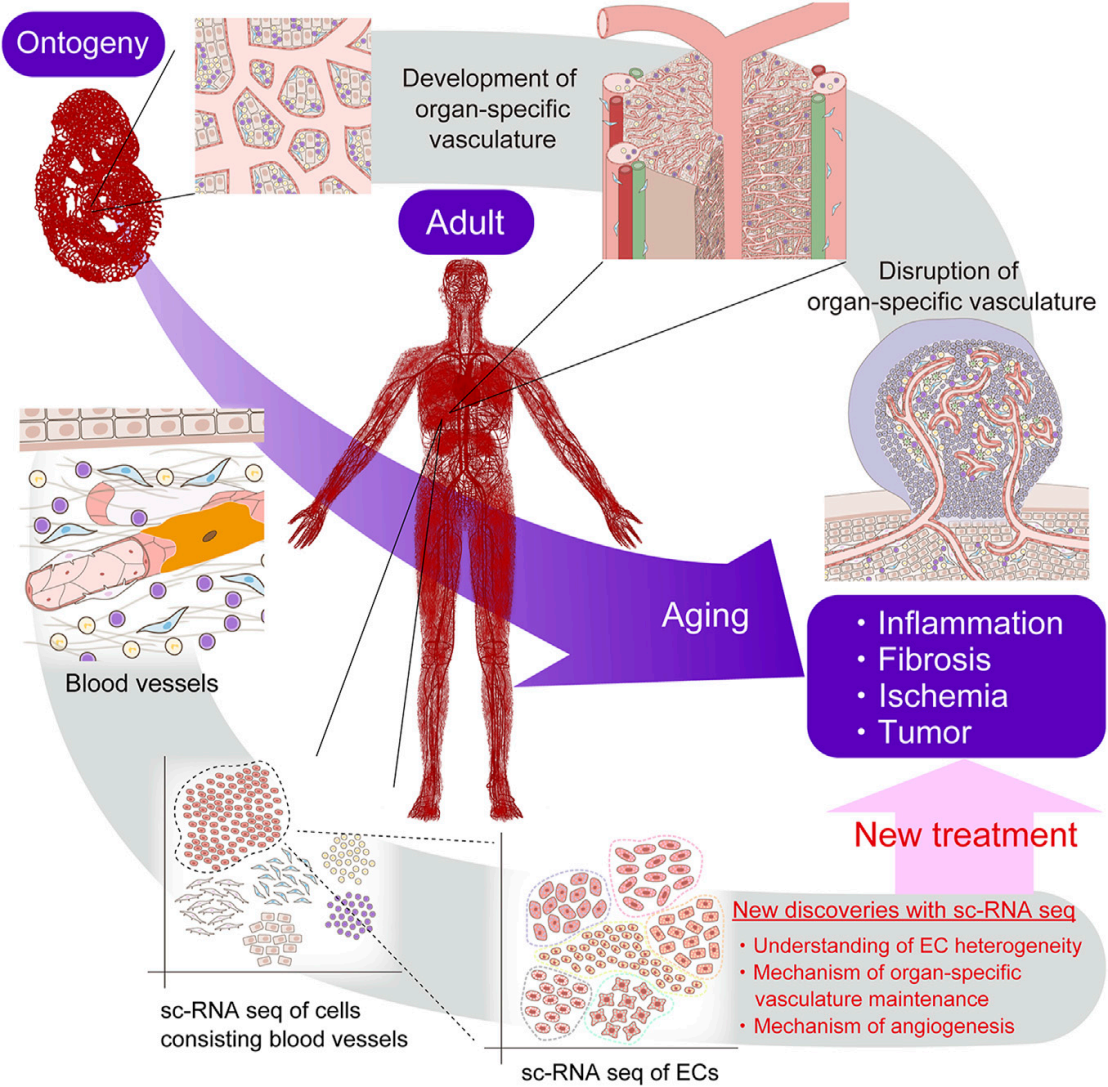
Future perspectives of single-cell RNA sequencing. Single-cell RNA sequencing is expected to elucidate vascular biological mechanisms and find new therapeutic targets.

The introduction of scRNA-seq technology has greatly improved our understanding of the highly diverse vascular endothelial cell heterogeneity in the context of vascular formation, organ homeostasis, and disease. Unlike bulk RNA sequencing, which has been used to study gene expression patterns at the cell population level, the ability to generate gene expression profiles at the single-cell level by scRNA-seq has enabled the discovery of specific EC cell types that exert unique functions in health and disease, thereby improving the understanding of the numerous roles of ECs in various tissues. scRNA-seq has also provided insights into vascular structure–functional relationships, gene regulatory networks, the hierarchy of ECs within the vasculature, and the roles of activated EC subtypes during pathological angiogenesis. In this article, we have discussed the EC heterogeneity across the vascular tree, including arteries, arterioles, capillaries, post-capillary venules, and veins. However, further heterogeneity may also exist within the same vascular bed; for example, within the ECs that form arteries. Further advances in single-cell analysis will hopefully provide even more detailed information regarding EC heterogeneity and EC subtype-specific responses to disease. Various single-cell omics technologies will help advance this analysis. The association between specific EC subtypes and physiological or pathological phenomena will be identified in the near future. Furthermore, a greater understanding of endothelial heterogeneity is expected to reveal the diversity within EC populations, while also identifying cellular communication with other cell types in health and disease. This information will be valuable for elucidating vascular biological mechanisms and finding new therapeutic targets (Figure 9). More importantly, single cell gene expression technology linked to the location of a cell in the tissue, known as spatial transcriptomics, may further enhance our knowledge (Rao et al., 2021; Chen et al., 2022). Anticipated new treatments developed by selective targeting of specific ECs may include drug delivery, revascularization, tissue-engineering, gene therapy, maintenance of BBB integrity for stroke and neurological disorders, and anti-angiogenic therapy targeting choroidal neovascularization and the tumor vasculature (Hennigs et al., 2021). Ultimately, these advances in treatment will likely benefit patients with many types of vascular diseases.
